# Transistors for Chemical Monitoring of Living Cells

**DOI:** 10.3390/bios8030065

**Published:** 2018-07-04

**Authors:** Benoît Piro, Giorgio Mattana, Steeve Reisberg

**Affiliations:** University Paris Diderot, Sorbonne Paris Cité, ITODYS, UMR 7086 CNRS, 15 rue J-A de Baïf, 75205 Paris CEDEX 13, France; giorgio.mattana@univ-paris-diderot.fr (G.M.); steeve.reisberg@univ-paris-diderot.fr (S.R.)

**Keywords:** transistors, organic electronics, transistor arrays, chemical sensors, cells monitoring

## Abstract

**Featured Application:**

**Animal testing will be soon replaced by better accepted and less expensive in-vitro cell culture models, which explains the recent demand for real-time cell culture monitoring systems. They can bring high throughput screening and could be used not only for biomedical purposes (drug discovery, toxicology, protein expression, cancer diagnostic, etc.), but also for environmental ones (qualification of pollutants cocktails, for example). Beyond this, in-situ monitoring also participates in strengthening the fundamental knowledge about cells metabolism.**

**Abstract:**

We review here the chemical sensors for pH, glucose, lactate, and neurotransmitters, such as acetylcholine or glutamate, made of organic thin-film transistors (OTFTs), including organic electrochemical transistors (OECTs) and electrolyte-gated OFETs (EGOFETs), for the monitoring of cell activity. First, the various chemicals that are produced by living cells and are susceptible to be sensed in-situ in a cell culture medium are reviewed. Then, we discuss the various materials used to make the substrate onto which cells can be grown, as well as the materials used for making the transistors. The main part of this review discusses the up-to-date transistor architectures that have been described for cell monitoring to date.

## 1. Introduction

Complex biological interactions or cellular stress responses difficult to highlight using conventional biosensors, can be identified because of the changes in cellular physiology. These changes are, typically, local changes in pH [1,2], in surface coverage of cells [3,4,5,6], in protein expression, or in cellular metabolism that induce local changes in the concentration of metabolites or biochemicals, such as glucose, lactate [7,8], dopamine [9], glutamate, acetylcholine, etc. Cell culture monitoring may also be applied to monitor cancer cells’ metabolism, for example.

Chemical sensing of cellular activity has been described for decades [10], particularly to study exocytosis, which is an important biological process used by cells to secrete molecules acting as messengers in their surroundings. Even if it is possible to use conventional carbon fiber ultramicroelectrodes to record exocytosis events, microfabrication techniques have significantly improved electrochemical sensing, particularly arrays of sensors [11]. For example, recent reviews have demonstrated that it is possible to record not only electrical cellular activity [12], but also to sense exocytosis events on conventional microelectrode arrays (MEA) [13,14]. Therefore, MEA may be used in basic research or clinical diagnostics for cell analysis–based drug discovery or environmental monitoring, where they help to monitor in-situ (in Petri dishes) not only cell functions via neurotransmitters release, but also enzymatic activity or cells respiratory activity (glucose consumption, lactate production), using electrochemical reactions on sensing electrodes [15].

Cellular responses to chemical stimuli must be, in turn, transduced into interpretable signals. Even if the optical characterizations of cell metabolism are commonly reported in the literature [16,17], direct electrical transduction is more attractive because the output signals are already in a form that will be directly usable in processing electronic circuits. Among the devices able to provide such electrical signals, we find electrochemical (potentiometric or amperometric) ones, which are most of the time used in MEA and will not be reviewed here, and transistors. Transistors present the advantage of transducing the recognition of (bio)chemical molecules directly into an electric signal. Moreover, such a signal is also amplified by the transistor effect. Several excellent reviews discussing the use of organic thin-film transistors for cell-based sensing have been published for a few years [18,19,20].

In this review, we will identify applications of silicon-based transistors and organic transistors. The latter are particularly promising candidates because they can work in aqueous media and physiological conditions, be low-cost, and have low-power consumption. Particularly promising are the organic electrochemical transistors (OECTs), which have recently attracted considerable attention for cell monitoring applications [21,22]. In most of the reported examples, transistors were fabricated with extensive use of clean-room photolithographic processes, which considerably increase the cost per device. In this review, we also address the problems of costs and portability by reviewing techniques such as inkjet-printing (IJP) fabrication and the utilization of plastic and paper substrates for cell-monitoring transistors. IJP has effectively demonstrated its capability of reducing fabrication costs, because it is a non-contact, material efficient, and digital technique that allows rapid device design and fabrication, in ambient conditions. Moreover, plastic substrates are much cheaper (still another factor contributing to costs reduction) and easier to transport and to store than glass and silicon, and may be more convenient for promoting cell adhesion.

In the first part, the various chemicals that can be sensed from living cells are reviewed. In the second part, we reviewed the substrate materials used for cells culture, which can be used as well for making the transistors. The following and main part discusses the various up-to-date transistor architectures that have been described for cell monitoring. In the conclusion, we will identify tracks for the further development of this field.

## 2. Chemicals to be Sensed 

There are many chemicals that can be monitored in a cell culture, among which are the components of the cell culture medium, which are necessary for keeping cells alive, and the metabolites, which are produced by the cells in basic conditions or after cellular stress response due to internal or external stressors. 

### 2.1. Components of Cell Culture Media

Among essential components of a cell culture medium, some are very generic, such as dissolved oxygen, glucose, or various ions, like Ca^2+^, Mg^2+^, Na^+^, K^+^, or H^+^ for pH monitoring. Some others are less generic, such as vitamins (e.g., vitamin B12), or amino acids, such as glutamine.

**Figure 1:**
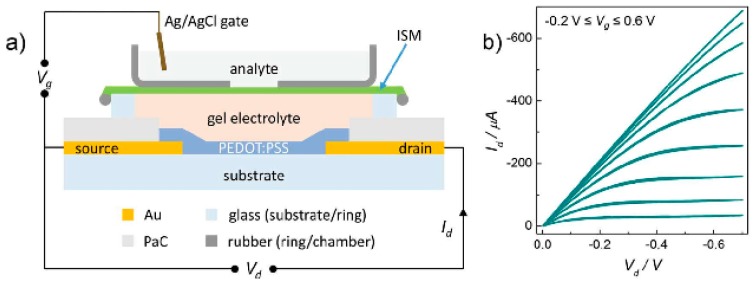
(**a**) Cross-section scheme of an ion-selective organic electrochemical transistor (IS-OECT) based on an ion-sensitive membrane (ISM), and (**b**) output curve of the device recorded in aqueous 10^−3^ M KCl. Reproduced from Sessolo et al. [27]. Copyright © 2014, John Wiley and Sons.

Oxygen quantification can be done by electroreduction on a Pt or Au electrode [2], based on a Clark electrode [23]. Glucose is typically detected using glucose oxidase (GOx), a redox enzyme that is highly specific for glucose oxidation, associated to an amperometric reaction that detects H_2_O_2_ (a side product of the enzymatic reaction) [24], or that electrochemically recycles an artificial redox mediator that replaces O_2_, the natural cosubstrate of the enzyme [25]. Glutamine can be detected through a very similar procedure, simply by replacing GOx with the enzyme glutamine oxidase (L-Glu), which also produces H_2_O_2_. Another way to detect glutamine is to use an enzymatic cascade with glutaminase, which produces L-glutamate from glutamine, followed by glutamate oxidase. Lastly, ions can be sensed using ion-selective devices such as ISFET-like devices, based on inorganic or organic materials [26,27,28]. Figure 1 shows an example of an in-between device, and an ion-selective organic electrochemical transistor (IS-OECT) [27]. Such transistors are, of course, among the most efficient devices for transducing the presence of a specific ion into a measurable signal, with a sensitivity significantly higher than using conventional potentiometric sensors. 

### 2.2. Metabolites

Among the metabolites of the cell cultures, one finds lactate, pyruvate, and L-glutamate, but also various neurotransmitters, biologically relevant ions, or other biomolecules [29].

**Figure 2:**
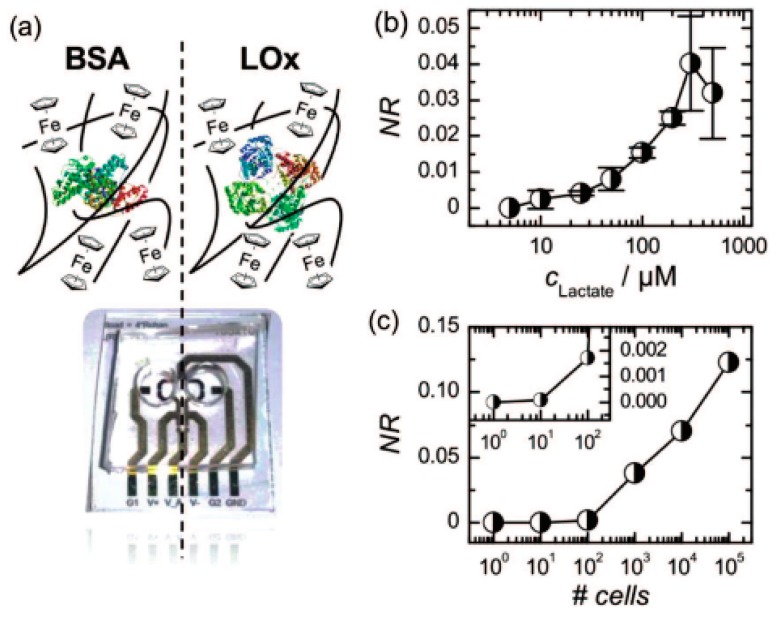
(**a**) Lactate oxidase and ferrocene cross-linked with chitosan and immobilized on the sensing organic electrochemical transistor (OECT) using epoxy terminated self-assembled monolayers. A second OECT is used as reference, where the same complex is used but where the specific enzyme is substituted by bovine serum albumin (BSA); (**b**) calibration curve for three different devices. The normalized response (NR) is NR = ∆V_out_/∆V_out,max_. The gate voltage is kept constant at V_GS_ = 200 mV; (**c**) titration curve for successive additions of media collected from cells cultured at different concentrations for 24 h. Reproduced from Braendlein et al. [7]. Copyright © 2017, John Wiley and Sons.

Lactate, pyruvate, and glutamate can be detected through a procedure very similar to that of glucose, simply by replacing the nature of oxidase enzyme, that is, GOx by LOx (lactate oxidase), PyOx (pyruvate oxidase), or GluOx (glutamate oxidase), respectively, all of these enzymes producing H_2_O_2_ from O_2_. For example, Braendlein et al. [7] described an OECT for the detection of lactate in a cell culture medium (Figure 2). One of the interesting features of this reported sensor is that the circuit provides an original inherent background subtraction based on the so-called Wheatstone bridge (invented in 1843, but very poorly used in the biosensors community until now). The Wheatstone bridge is based on the balance between two legs of a bridge circuit, one leg of which includes the reference transistor, and the other leg being the sensing transistor; the balance is very sensitive to changes in the resistance of one of the two legs, which provides an extreme overall sensitivity.

Neurotransmitters other than L-glutamate are more difficult to detect because there are no specific enzymes. For example, epinephrine [30] or dopamine [31] can be easily electrooxidized, at relatively low potentials, but numerous other biomolecules (the archetypal one being ascorbic acid) can be also oxidized at the same low potentials, which prevents this approach from being selective. The same difficulties arise for other metabolites that can be found in culture media, but specific biosensors have been extensively reported (DNA [32], proteins [33], hormones [34], carbohydrates other than glucose [35], steroids [36], or even antibiotics [37]). Such biosensors were exclusively based on classical transduction methods (e.g., amperometry) and actually very few are based and transistors.

## 3. Substrates for Cell Culture 

### 3.1. Approaches for Cells Localization

Biological assays are most often based on cell populations instead of a single cell, which allows for averaging the cellular responses but loses the temporal data. For electrical or electrochemical sensors, another objective for cell monitoring is to force cells to set close to the sensing electrodes, so that the released products (e.g., protons, metabolites, proteins, or catecholamines) do not have to travel a long distance, being diluted, before being detected. These issues have been solved by trapping single cells (or at least a few cells) close to the measuring electrode. Several approaches for making structured substrates, such as microarrays of wells or cellular patterns, can be achieved by surface modification, where the patterns of biomaterials support or inhibit cell adhesion, using photolithography, dip-pen nanolithography, scanning probe lithography, microcontact printing, etc. Adherent cells (most cells) can be localized on pre-defined areas of a culture surface using chemical or physical patterning methods [38]; on the contrary, non-adherent cells (e.g., stem cells) can be localized using techniques such as negative dielectrophoresis [39] or etched microwells [40]. Reviews dealing with microwell fabrication methods for cellular studies are available [41]. These approaches can be divided into physical and chemical methods, which may also be described as passive and active. Both are pertinent, but one may bear a particular interest to the active methods able to place, move, or remove cells on the sensing substrate. These active methods include the printing of cells on substrates, including with contactless inkjet printing, which is very promising. 

#### 3.1.1. Passive Methods 

The passive confinement of cells in microwells is attractive for its simplicity [42,43,44]. The reader who wants to go into detail can read specific reviews that have been previously published [45,46,47].

**Figure 3:**
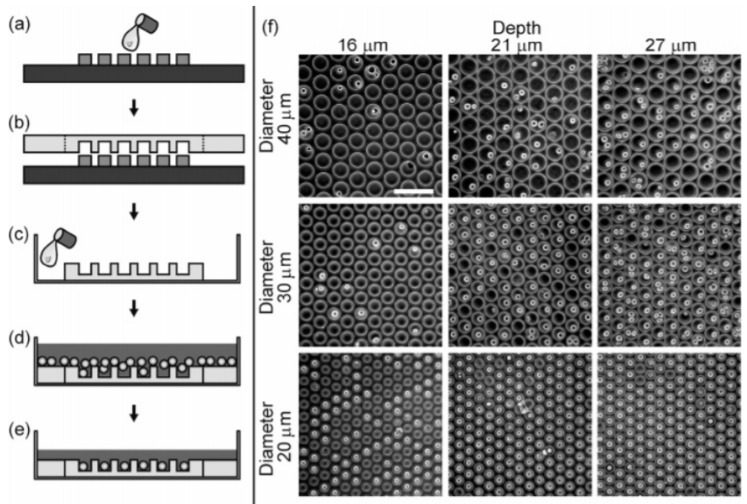
Cells trapped in polydimethylsiloxane (PDMS) microwells. (**a**–**e**) Fabrication and seeding procedures (photolithographied master and PDMS, peeling-off, PDMS wells peeled off and put at the bottom of a Petri dish, cells settlement into the peeled-off wells); (**f**) cells remaining in microwells, depending on their diameters and depths. Scale bar: 100 µm. Reprinted with permission from Retting et al. [43]. Copyright © 2005 American Chemical Society.

For example, a simple method for controlling the spatial positioning of cells and bacteria on substrates was described by Koh and colleagues [48], making microwells trapping cells composed of hydrophilic polyethyleneglycol (PEG) walls and hydrophobic 3-(trimethoxysilyl)propyl methacrylate bases, made on various substrates such as silicon, glass, or polydimethylsiloxane (PDMS) surfaces. The same kind of approach was used by Rettig et al., where a microwell array was made by photolithography and then PDMS molding, as described in Figure 3 [43].

#### 3.1.2. Active Methods

There are various non-physical methods to confine cells or at least to control their development on defined areas of a substrate. These methods can be dynamic (i.e., triggered by an external stimulus). For example, Bolin et al. demonstrated that epithelial cells seeded on the channel of electrochemical transistor made of poly(3,4-ethylenedioxythiophene):tosylate (PEDOT:TOS) can grow, depending on the potential gradient along the channel. In other words, the cell population along the channel is controlled by the gate and drain potentials [49]. Even if it is not as mature as electrowetting, this approach is particularly interesting because the transistors and organic semiconductors here are not only being used for measuring, but also for as actuators.

**Figure 4:**
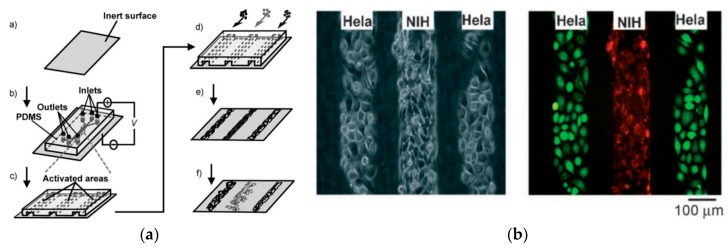
(**a**) Strategy for patterning cells. a) Self-assembled monolayers (SAMs) are formed on a gold-coated surface; b) stamping, followed by applying a microfluidic PDMS cover; c) application of a cathodic potential on the gold substrates to desorb the SAM; d–f) adsorption of proteins followed by adhesion of cells. (**b**) Phase-contrast (left) and fluorescence (right) micrographs of cells patterned as described above. Adapted from Li et al. [52]. Copyright © 2007, John Wiley and Sons.

Electrowetting was also proposed. It is a technique that is able to move microdroplets by applying an electric field that changes their contact angle on the polarized surface; cells can be transported inside of these droplets. Azam Shaik et al. proposed an array of thin film transistors able to dynamically polarize a surface into reconfigurable patterns. They applied this technique to move HepG2 carcinoma cells (a human liver cancer cell line) on a surface [50]. Electrochemical patterning has been also investigated, for example, based on the self-assembled monolayer of alkanethiolates on gold, that they could be switched from a state that prevents the attachment of cells to a state that promotes it [51]. Another approach is the selective electrochemical desorption of an anti-adhesive self-assembled monolayer. Li et al. used this strategy in microfluidic channels, for the adhesion of several types of cells in a controlled geometry (Figure 4) [52].

**Figure 5:**
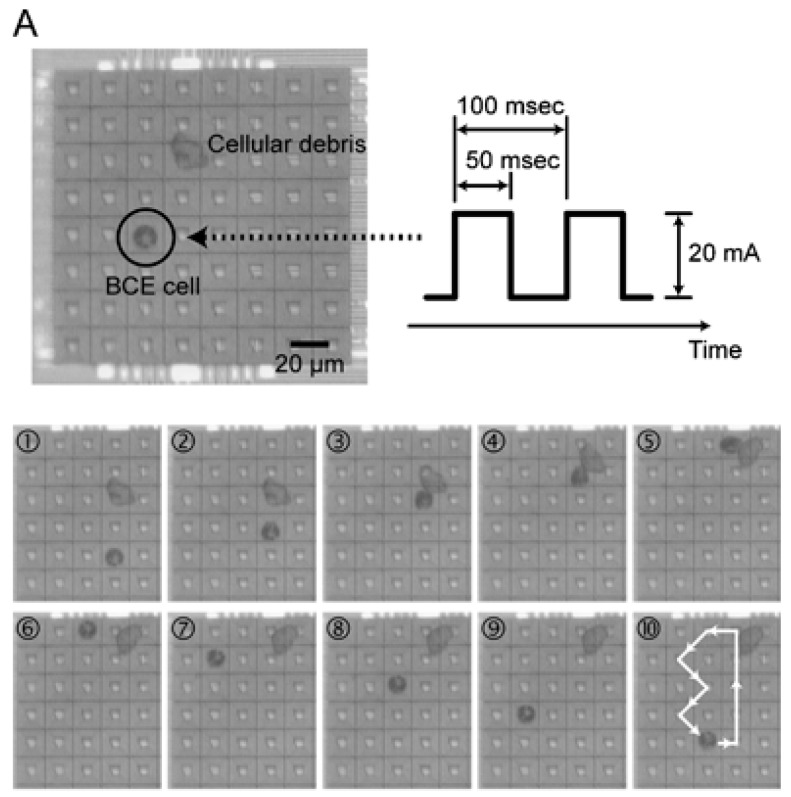

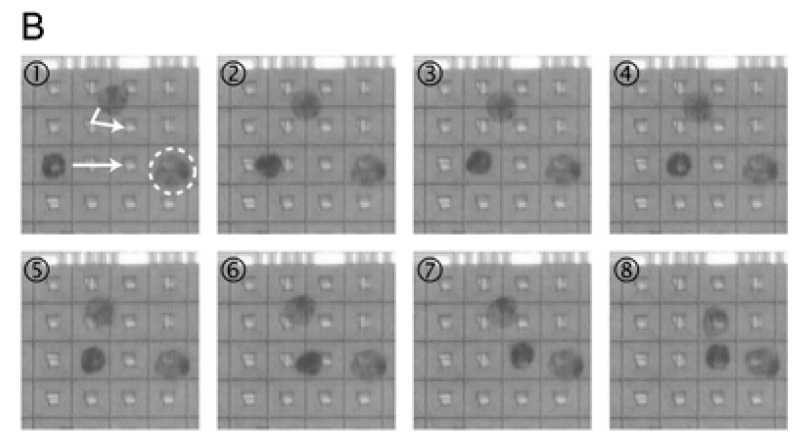
Manipulation of bovine capillary endothelial (BCE) cells. (**A**) A single BCE cell trapped on the center of a microcoil (current generating the magnetic field: 20 mA of amplitude, with 50% of duty cycle). The cell is then moved over the array to make a round trip. (**B**) Manipulation of three cells. The cell inside the dotted circle is trapped and kept on the corresponding microcoil, while the two remaining cells are moved toward it. Reproduced with permission from Lee et al. [53]. Copyright © Royal Society of Chemistry, 2007.

Also, in 2007, Lee et al. [53] demonstrated the possibility of using a standard CMOS (complementary metal-oxide semiconductor) chip coupled with a microfluidic system specifically designed for cell manipulation. The microfluidic part of the chip was equipped with a temperature regulation system to provide ideal biological conditions for cell growth; very interestingly, the upper part of the chip was supplied with an 8 × 8 microcoils array, and each microcoil was electronically controlled by the CMOS chip and able to locally generate a magnetic field. On the top of the system, a layer of bovine capillary endothelial (BCE) cells was grown; before deposition on the chip, the cells were incubated in a solution containing peptide-coated magnetic beads, which were absorbed inside the cells cytoplasm by endocytosis. The authors thus demonstrated that by activating a single microcoil, the interaction between the magnetic field and the bead contained inside the cells was strong enough to block the cell onto the active microcoil; moreover, by switching on sequentially adjacent microcoils, it was possible to move the cells along the substrate, as shown in Figure 5.

#### 3.1.3. Printing

The idea for printing MEAs is not new; one of the first examples was given by Seddon et al. in 1994 [54], even if not for cell monitoring, but for the detection of heavy metal traces. The array was made of a network of several thousands of 15 μm diameter carbon disc microelectrodes over an area of 5 mm × 5 mm, and they separated one another by a few hundreds of micrometers. The fabrication process relied on a conventional thick-film printing technique, followed by local photoablation.

The microwells can be printed; however, another approach consists of printing primary layers that promote or, on the contrary, prevent cell adhesion, for example, by inkjet printing (IJP) of collagen solutions, which help cellular attachment and proliferation on a scaffold [55]; IJP of Poly(lactic-co-glycolic acid) (PLGA) [56] or PEG hydrogels [57,58]. In an attempt to merge the two approaches (printed wells and printed cells), Liberski et al. [57] described a very interesting approach to prepare the arrays, not by the IJP of solid wells, which are then filled by cells, but by printing first an array of cell culture medium droplets on the substrate, then printing on top of it a hydrophobic thin layer of mineral oil over the droplets, then printing the cells into the culture medium droplet. An excellent review [59] was published in 2018 by Feng et al., who discussed not only the droplet deposition, but the reactions between droplets (e.g., cellular communication in-between droplets).

Beyond the printing of microstructured substrates for cell immobilization, the direct printing of cells is also described. These approaches were reviewed, for example, in Barbulovic-Nad et al. [60], in particular, contact printing and non-contact printing, the latter being photochemical methods, such as laser writing, electrospray deposition, but also inkjet technologies. For example, Fujita et al. [61] proposed cell microarrays made by the inkjet printing of plasmid and extra-cellular matrix protein on a hydrophilic substrate (glass treated by polyethylene glycol). Spots were of 50 μm in diameter, separated by 150 μm. Considering that printing may destroy fragile cells, such cell microarrays have also been obtained in another way [62], by pre-functionalizing the substrate by albumin as a hydrophilic agent, followed by spotting polyethyleneimine (PEI). Cells grown on this substrate only adhere on the PEI-modified spots. However, more recent works have shown that the IJP of viable cells is also possible [63]; because nozzle clogging generally occurs when multiple cells are ejected from the nozzle, the jetting of individual cells is preferred.

All of these approaches, direct printing of cells or printing of scaffolds, are summarized in the book of Tse et al. [64]. 

### 3.2. Application of Cells Localization to Electrochemical Sensors, then to Transistor-Based Sensors

**Figure 6:**
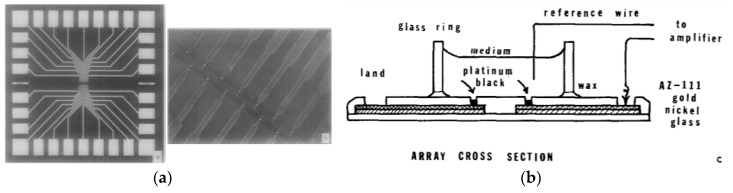
One of the first examples of microelectrode array. (**a**) Left: full photolithographied array and its contacts; right: zoom-in of the arrayed platinum black electrodes forming the floor of the well; (**b**) scheme of the device. Reprinted from Thomas et al. [65]. Copyright © 1972, with permission from Elsevier.

In 1972, an article already described a microelectrode array (MEA) [65] for recording the electrical activity of heart cells, even if, in this pioneering work, the localization of the cells was simply obtained by using a macroscopic glass chamber (Figure 6).

**Figure 7:**
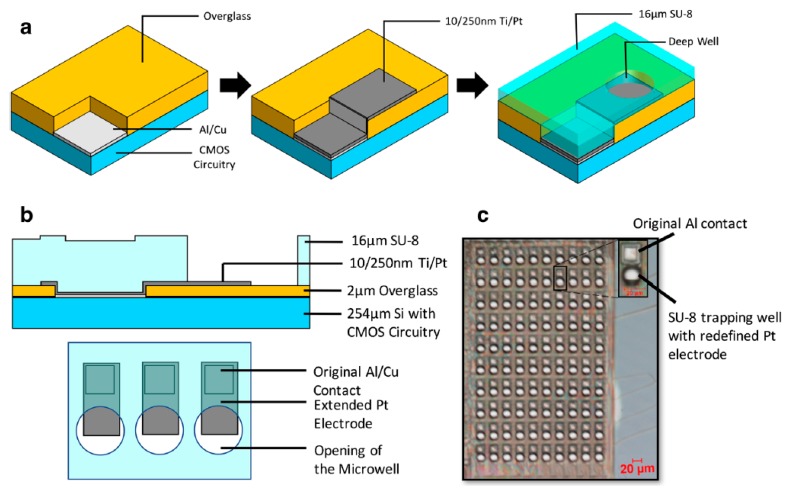

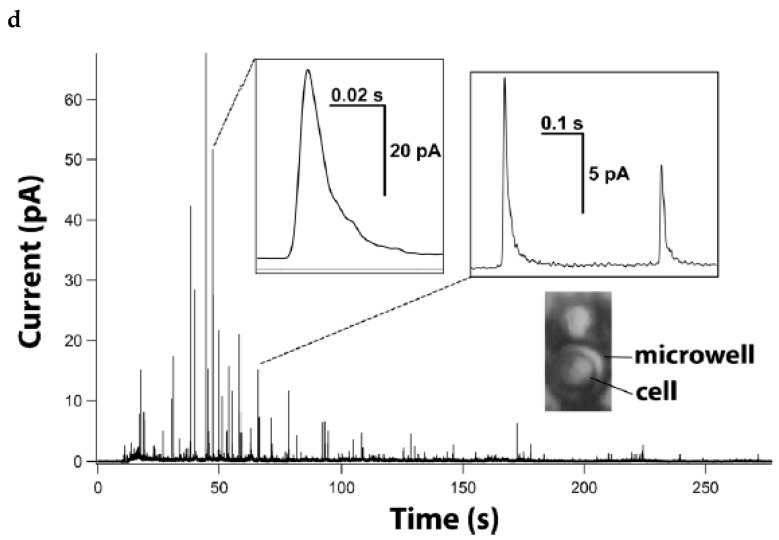
(**a**) Fabrication process of the microwells by photolithography; (**b**) side view of the microwell structure; (**c**) micrograph of a sensor array (300 × 500 μm); and (**d**) signal recorded for a unique live chromaffin cell settled down in a well, in direct contact with the electrode. Adapted from Huang et al. [14]. Copyright © 2017 Springer-Verlag GmbH.

MEAs for the electrochemical (most often amperometric) detection of cellular activity using photolithographied wells for single cells (or groups of a few cells) have been described since 1976 for oxygen quantification [66]. Amperometry is used, for example, to measure electroactive neurotransmitters such as epinephrine, dopamine, or catecholamine, by the electrooxidation of these molecules at a sufficiently high potential. If these molecules are released by the cells (e.g., at the occasion of exocytosis events), each release produces amperometric spikes. These spikes reveal the release kinetics on the level of a single exocytosis event, provided that the signal is laterally resolved (i.e., not convoluted with the releases of other neighboring cells), and also is time-resolved (i.e., does not suffer from diffusional broadening, which happens when the release area is far from the detecting electrode). To allow a statistical analysis of these exocytosis events, a large number of release events from a large number of cells must be measured locally and simultaneously. For efficient targeting of single cells specifically to the electrode sites, the most common approach consists of patterning the microwell structures [14] (Figure 7). These approaches have been reviewed recently by Ino et al. [67].

## 4. Devices

This section reviews the various transistor-based devices that have been reported for ex-situ (cell culture media are brought to the sensor) or, much more interestingly, in-situ (sensor active within cell culture media) monitoring of cells activity. Firstly, inorganic transistors are reviewed (field-effect transistors or liquid-gated transistors), then, organic transistors, including the electrochemical ones that represent the vast majority of the reported works.

### 4.1. Inorganic Transistors

#### 4.1.1. Field Effect Transistors

The basic concepts concerning the field effect are applicable, under certain conditions, to both inorganic and organic transistors. It is usual to cite the patent filed by Julius Edgar Lilienfeld in 1930 [68] as the very first description of a field-effect transistor (FET), in contrast with the later work of J. Bardeen, W. Shockley, and W. Brattain, who claimed its invention in 1947 and who received the Nobel Prize in Physics in 1956 for their work.

**Figure 8:**
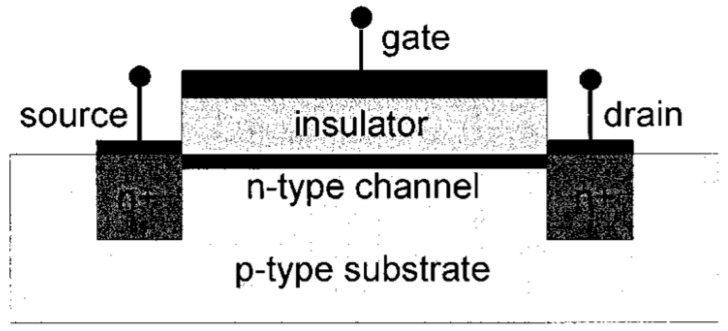
Schematic views of the metal/insulator/semiconductor fet (MISFET), also called MOSFET when the insulator is silicon oxide. Adapted from Horowitz [69]. Copyright © 1999, John Wiley and Sons.

An FET usually comprises four electrodes, namely: body, source, drain, and gate electrodes; considering that the body and source are generally short-circuited, it is usual to consider the three latter contacts only. A semiconducting channel sits between the source and drain. The density of the charge carriers (electrons or holes) in the channel, responsible for the current flow, is modulated by the gate voltage. Long after Bardeen, Shockley, and Brattain’s work, the first silicon-based FET was described in the 1960s, which is still the actual form of today’s transistors (Figure 8, extracted from the review of Horowitz, 1998 [69]). For an n-type FET working in the enhancement mode of operation, upon the application of a sufficient positive voltage to the gate, an n-type channel forms at the insulator/semiconductor interface ,where the electron density becomes sufficient to allow a current of electrons to flow between the source and the drain. This turns the device on. For a p-type FET, the applied gate voltage should be negative, and the charge carriers are holes instead of electrons.

●  Ion-sensitive field effect transistors

**Figure 9:**
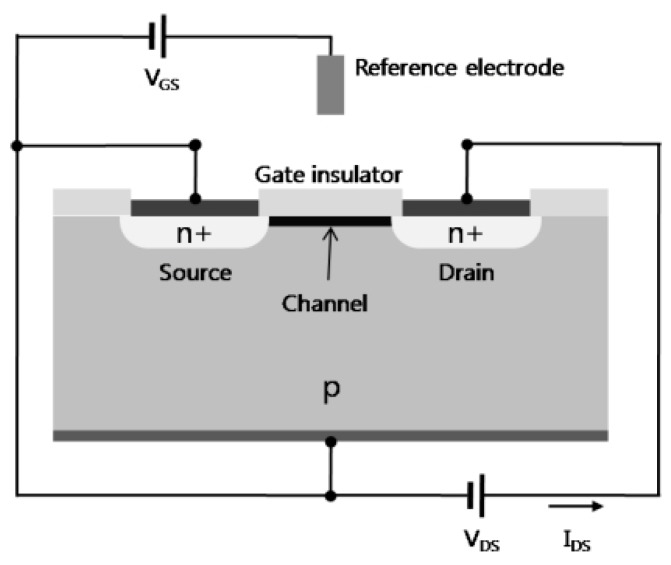
Structure of an ion-sensitive field-effect transistor (ISFET): source, drain, gate insulator, reference electrode, electrolyte in-between the reference, and the gate insulator. Reproduced from Lee et al. [70], under Creative Commons Attribution License (CC BY 3.0).

Maybe the most common FET architecture used in biosensors to date is the ion-sensitive field-effect transistor (ISFET). This device is made of a reference electrode that also serves as gate electrode dipping into an electrolyte, which is in direct contact with a dielectric deposited over the semiconductor. In this configuration, the drain current is driven by the potential of the electrolyte–insulator interface, which is the sensing interface (Figure 9). Therefore, the insulator must be functionalized in order to be sensitive to a given analyte. The most common ISFET is sensitive to protons, for example, by using silicon nitride. With this material, however, the operating gate potentials are high (several volts). To lower down the gate potential, other dielectric materials are used, for instance tantalum oxide (Ta_2_O_5_), which provides a higher capacitance than silicon nitride and therefore allows lower operating potentials. The review of Lee et al. [70] is available for readers who would like to have more insights on the ion-sensitive field-effect transistor for biological sensing.

Ji et al. developed, in 2008, an ion-sensitive field effect transistor (ISFET) made on a flexible substrate (polyethylene naphthalate) to measure the concentration of potassium cations in a physiological medium. Valinomycin, a naturally occurring molecule that is involved in the potassium ion transport processes in cells, was used as the ionophore and was immobilized on the gate electrode. One may consider that this work opened the way to the development of living cells-based ISFETs [28]. Zhu et al. described, more recently, the fabrication of a graphene-based ISFETs for measuring the K^+^ production from glial cells. The device was fabricated by modifying the gate electrode by graphene and valinomycin, which guaranteed specificity for K^+^ without interference with Na^+^ or even Ca^2+^. The sensor was brought close to the rat glial cells. The activation of the K^+^ channels led to a shift of the transfer curves toward more positive potentials [71]. This work is interesting because it shows that ISFETs are intrinsically sensitive to ions, without specificity; this specificity must be brought by an external element. The other interesting feature is that the gate potential, which triggers the current flow through the transistors, can also trigger the ion flux through biological ion channels, which fully exploits the ion-to-electron transducing feature of these transistors and also paves the way for solution-gated ones (developed in Section 4.1.2).

**Figure 10:**
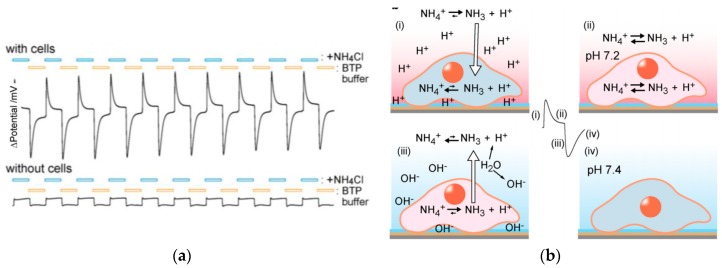
(**a**) Time-course of the ISFET potential difference (between gate and drain) during periodic flushes (60 s each) of isotonic buffers containing 10^−2^ mol L^−1^ NH_4_Cl, or 2 × 10^−2^ mol L^−1^ sucrose. A sharp pH change occurred when the buffer solution surrounding the cells on the gate insulator was exchanged in a stepped manner. pH transients were not observed when no cells were present on the gate insulator. (**b**) Illustrations of the mechanism. Reproduced from Imaizumi et al. [72]. Copyright © 2017, with permission from Elsevier.

In order to monitor the membrane integrity of the living cells, which could be challenged by cytotoxic chemicals, Imaizumi and coworkers described a pH-sensitive ISFET able to measure the time-resolved changes in pH, generated by the HepG2 living cells immobilized on the gate insulator of a proton-sensitive FET, when these cells are subjected to flushing with NH_4_Cl. The cell activity decreased when a cytotoxic molecule was added [72]. The ISFET used a p-type Si as semiconductor, SiO_2_ as dielectric, and Ta_2_O_5_ as gate-sensitive material, pre-treated with Poly-l-lysine. The living cells were directly immobilized on the gate in a 5 mm diameter well. Figure 10 shows the potential response of the device upon the addition of a 1,3-bis(tris(hydroxymethyl)methylamino)propane (BTP) buffer and NH_4_Cl, or a BTP buffer and sucrose. For these measurements, the ISFET was operated as a source-drain follower (i.e., with a constant drain-source voltage of 0.5 V, a constant drain-source current of 0.5 mA, and no DC bias potential against the Ag/AgCl gate electrode). The output signal was the voltage between drain and reference.

**Figure 11:**
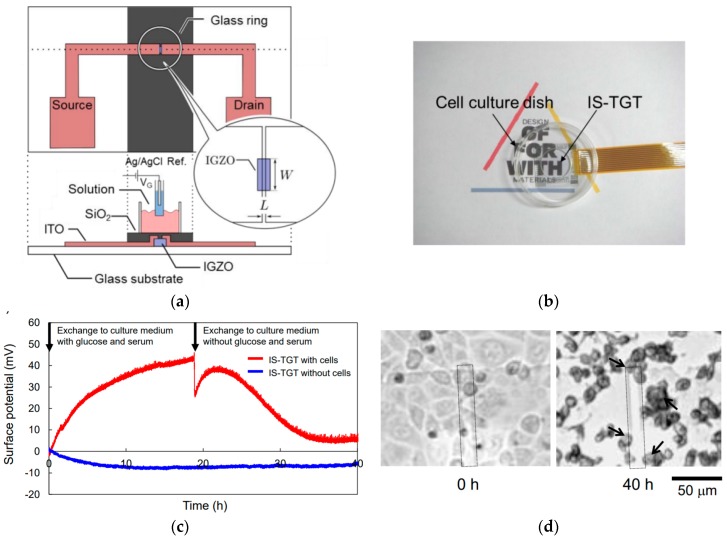
(**a**) Scheme of the ion sensitive transparent-gate transistor (IS-TGT) structure; (**b**) image of a cell culture dish with IS-TGT; (**c**) change in surface potential measured by the IS-TGT with HeLa cells in the well; and (**d**) optical micrograph of living cells (0 h) and dead cells (40 h). The gate area is shown by the dotted line. Adapted with permission from Sakata et al. [2]. Copyright © 2017 American Chemical Society.

To follow the chemical activity of the living cells while being able to observe them using an optical microscope, Sakata et al. developed a transparent ISFET, which they called ion sensitive transparent-gate transistor (IS-TGT). The semiconductor was made of In–Ga–Zn-oxide (IGZO), over which a thin SiO_2_ film was deposited as a gate dielectric. The cell activity was monitored simultaneously with microscopic observations and electrical measurements, with a clear dependence on the gate potential with pH, K^+^, and Na^+^ concentration [2] (Figure 11).

●  Other kinds of classical inorganic field-effect transistors

Besides ISFET devices, classical MOSFETs have been described for the monitoring of cell detachment. For example, Nabovati et al. [3] reported a capacitive sensor array using the CMOS technology for in-situ cell growth monitoring. They constructed an array of 8 × 8 CMOS that measured capacitance at the interface between the sensor and the cell culture medium. Practical results were obtained with H1299 cells (human lung carcinoma cell). This report is very recent; there is, to our knowledge, no other example of CMOS-based living cell monitoring systems at the moment, but one may expect a quick development of this area in the near future.

#### 4.1.2. Electrolyte-Gated Field-Effect Transistors

Electrolyte-gated field-effect transistors (EGFETs) have a very similar architecture compared to ISFETs, but there is simply no solid dielectric on the semiconductor, and the gate electrode is not necessarily a reference electrode, but can also be a bare metal. In an EGFET, upon the polarization of the gate, an electrical double layer (EDL) is formed at the gate/electrolyte and electrolyte/semiconductor interfaces. These double layers, and more particularly the electrolyte/semiconductor EDL, act as a dipole of a few angstroms of thickness only, which creates an extremely strong electrical field across the interface; this electric field is finally able to attract mobile charge carriers from the semiconductor bulk to its surface, thus creating a highly conductive, thin layer between the source and drain electrodes. It should be noted that any change in terms of surface potential (on the gate or on the semiconductor) will drive a change in the drain current. 

One of the earliest reports dealing with EGFET for cell monitoring, Steinhoff et al., described an AlGaN/GaN-based electrolyte gate FET array for the detection of extracellular electrical potentials. They reported the recording of extracellular action potentials from a confluent layer of rat heart muscle cells put directly on the gate, with a signal amplitude of 75 mV (signal-to-noise ratio of five) [73].

**Figure 12:**
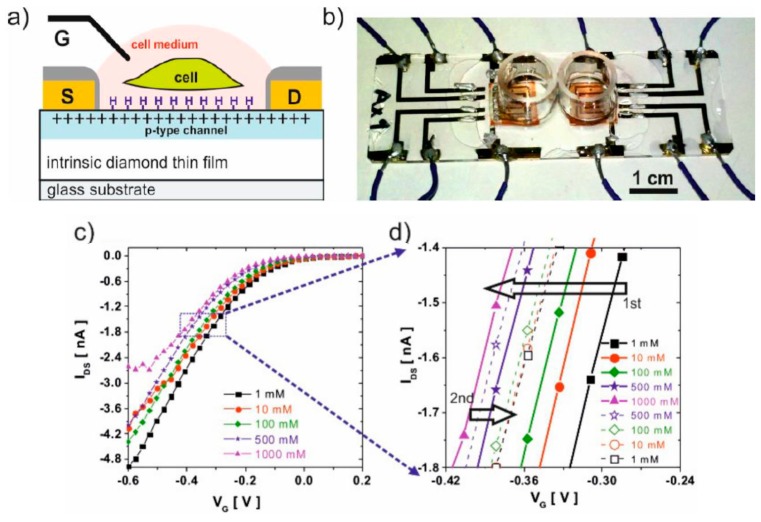
(**a**) Schematic view of the diamond-based electrolyte-gated FET (EGFET). Polarization of the gate/electrolyte interface creates a conductive p-channel in the intrinsic diamond semiconductor; (**b**) real device showing two wells above two sensors; and (**c**,**d**) transfer curves of the EGFET as a function of sodium concentration. Reproduced from Ižák et al. [74]. Copyright © 2017, with permission from Elsevier.

Similarly, transparent EGFETs have been also utilized for cells monitoring. For example, Izak et al. proposed real-time monitoring of cell growth through a diamond-based electrolyte-gated FET sensitive to pH, Na^+^, and K^+^, as well as to the adhesion of cells. As explained above, the difference between this EGFET configuration and an ISFET configuration, is that the diamond gate was free of gate oxide, which brings a higher sensitivity for small potential changes above the gate electrode [74]. In this work, the cellular activity was followed by changes in the threshold voltage of the device (Figure 12). A similar EGFET configuration was proposed by Procházka et al.; they studied yeast cells on their device and related the cellular metabolism with changes in potential (mostly because of pH changes) over the gate electrode [75].

**Figure 13:**
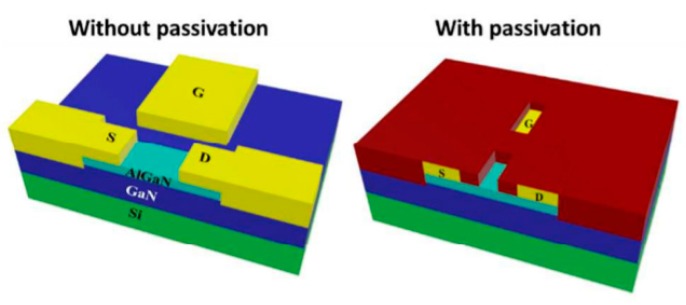
Structure of the electrical double layer FET (EDLFET), using AlGaN as a semiconductor, with and without the passivation layer (to better see the structure). Adapted from Pulikkathodi et al. [76], with permission of The Royal Society of Chemistry.

A very impressive work was also published by Pulikkathodi et al. in 2018 [76]. The authors developed a methodology where the EDL formed on a metallic gate electrode was used to modulate the channel conductivity of an electrical double layer FET (EDLFET). As shown in Figure 13, after insulation, only a part of the channel and the gate remain in direct contact with the surrounding solution. If optimized, this tiny hole can trap a few cells, which are then in contact with the gate. 

**Figure 14:**
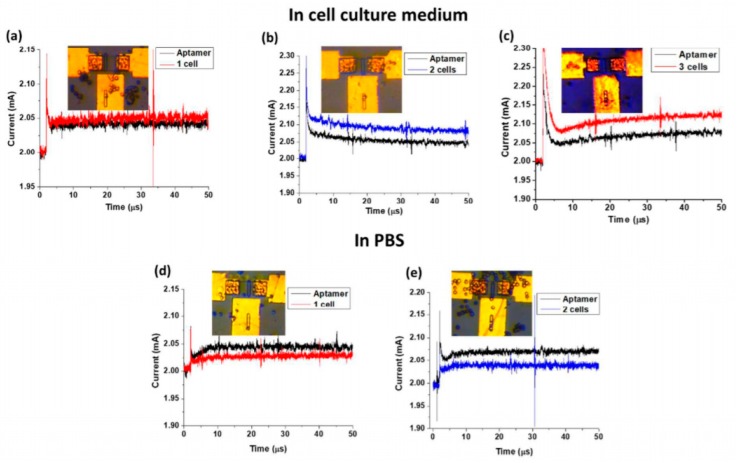
Detection and enumeration of circulating tumor cells in culture medium (**a**–**c**) and in phosphate buffer saline (PBS) (**d**,**e**). The cells captured on the device are clearly visible as brown circles. Adapted from Pulikkathodi et al. [76], with permission of The Royal Society of Chemistry.

The authors applied their device to the enumeration of circulating tumor cells and investigated their bioelectric signals, achieving a single cell resolution. To achieve this result, they functionalized the well with a DNA aptamer specific for the cells. As shown in Figure 14, one, two, or three cells can be trapped in the well and can modify the drain current accordingly. With this device, it is possible to investigate bioelectric signals from cells, with a high sensitivity (if the changes in the input signal are low, the output signal is high, of several tens of μA, which is easily measurable). Clearly, it is possible to monitor the changes in the transmembrane potential due to the opening or closing of the embedded ion channels. 

### 4.2. Organic Transistors

The organic field-effect transistors’ working principles are similar to those of the inorganic ones, given in Section 4.1.1. It is not the purpose of this review to go into the details of the charge transport within the organic conjugated materials, but the readers who want to go into deeper details can read the article of Horowitz, 1998 [69], or Coropceanu et al., 2007 [77]. In the category of OFETs designed for the cells monitoring, one finds ISOFETs (ion-selective OFETs) and EGOFETs (electrolyte-gated OFETs). The other category is the organic electrochemical transistor (OECT). 

#### 4.2.1. Organic Electrochemical Transistors

As for the other devices, it is first necessary to recall the functioning principles driving the organic electrochemical transistors (OECTs). They present a very similar architecture compared to EGFETs, with the difference that the semiconductor is organic and that the ions, instead of forming double layers at the gate/electrolyte and electrolyte/semiconductor interfaces, penetrate inside the materials (i.e., inside the organic conducting material forming the channel). This means that the basic working principle of OECTs relies on the reversible electrochemical doping of the channel, upon the application of an appropriate gate voltage. The material constituting the channel is generally Poly(3,4-ethylenedioxythiophene) doped with Poly(styrene sulfonate) (PEDOT:PSS) [78,79], a p-type conducting polymer. Without a gate bias, the polymer is in its conducting state and a current can flow between the source and drain. This is also true if the gate electrode is negatively polarized with respect to the source contact, the channel being maintained in its oxidized form. But if a positive gate voltage is applied, reduction occurs at the channel, which is consequently de-doped and becomes less conducting. More details are given, for example, in the article of Nielsen et al. [79], or the review of Rivnay et al., published in 2018 [78].

●  Ex-situ monitoring 

OECTs have demonstrated their applicability for sensing ions [80], pH [81], metabolites such as glucose or lactate [7,82], or neurotransmitters [32,33]. Again, these applications have been recently reviewed [21]. More precisely, Strakosas et al. [82] described the immobilization of lactate oxidase and glucose oxidase enzymes in a hydrogel at the platinum gate of an OECT, and evaluated their sensing ability by tracking their glucose consumption and lactate production, ex-situ, in a cell culture medium that was treated, or not, with cisplatin. This work showed the possibility to monitor metabolite concentrations, and more particularly, the ratio between the two metabolites’ concentrations, to predict the response of living cells under a given stress (e.g., upon the presence of toxic compounds). The experimental setup, using cells as a filtering membrane, was original. M. Braendlein et al. ([7]) described a similar device, still ex-situ, used to monitor an ensemble of 10^5^ cultivated cells (peripheral blood mononuclear cells), which produced an estimated lactate concentration of 10^−5^ M after 24 h in their experimental conditions. More importantly, they pointed out a clear difference in activity between normal and cancer cells, confirming the enhanced glycolytic metabolic activity of the latter.

●  In-situ monitoring 

As reviewed above, most of the devices rely on the measurements made from solutions picked up from cell culture, and are not in-situ. Even if providing a unique insight into the possible transductions that can be used to monitor living cells, ex-situ monitoring cannot compete with in-situ monitoring. To achieve this, the approach should consist of cultivating cells directly on the substrate where transistors are fabricated, and as close as possible to the gate of the OECTs, used as the sensing electrode by way of proper functionalization. 

**Figure 15:**
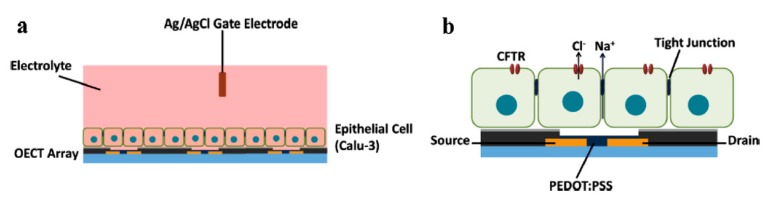
Human airway epithelial Calu-3 cells on top of an OECT array. (**a**) Architecture of the device. Cells form a dense monolayer on top of the surface, in-between the OECTs and the electrolyte; (**b**) detail showing the ion exchange occurring through the cell monolayer. Reproduced from Yao et al. [83]. Copyright © 2013, John Wiley and Sons.

Yao et al. described, in 2013, the architecture of an OECT-based cell monitoring system, where a monolayer of an epithelial cell was cultured on the semiconductor surface of OECTs. As shown in Figure 15, the cells established a polarized monolayer with the apical side facing the electrolyte and the basolateral side attached on the substrate. It was supposed that tight junctions between the cells could restrict the passage of ions from the electrolyte to the semiconductor. The activity of the cells, more particularly the opening and closing of cystic fibrosis transmembrane conductance regulators (CFTR, nanopores controlling the transepithelial chloride flow), also controls the sodium flow and more particularly, its sodium concentration at the basolateral side close to PEDOT:PSS. This change in ionic strength results in a change in the electrical characteristics of the transistor [83]. Again, as described in Strakosas et al. [82], an ensemble of cells was used as a membrane. This setup is able to control, depending on how tight cells are on top of the electroactive material, whether te cells are healthy or not. 

**Figure 16:**
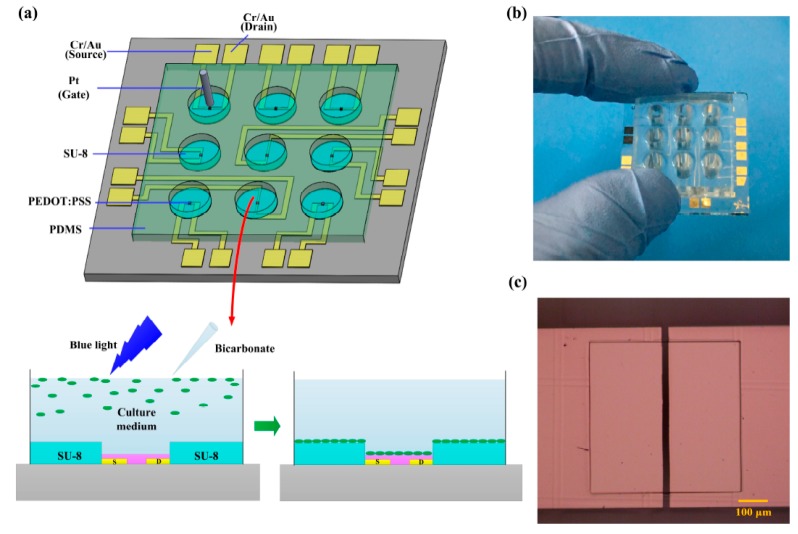
(**a**) Scheme of an OECT array. The electrical characteristics of the OECTs depend on the presence of the *H. pluvialis* cells, whether in the solution or adherent to the transistor; the culture medium serves as the electrolyte; (**b**) photograph of the OECT array platform; and (**c**) micrograph of the SU-8 microwell. Reproduced from Wei et al. [4] under the Creative Commons Attribution License (CC BY 3.0).

Salyk et al. described an array of 96 wells at the bottom of which sat OECTs having a channel (fabricated in PEDOT:PSS) of 1.5 mm^2^ surrounded by a circular gate electrode, onto which the electrogenic cells (3T3 fibroblast cells) were cultivated. Because of the extensive ion exchange, the cells growing at the transistor channel modulated the current within it; hence, the removal of cells resulted in changes of the channel current, thus making it possible to distinguish between the wells occupied by living cells from those unoccupied, or from the wells occupied by dead cells [84]. A similar work was made with unicellular microalgae (Haematococcus pluvialis) settled on a PEDOT:PSS OECT. This alga is known to produce an interesting metabolite, astaxanthin (a high-value anti-oxidant), which is laborious to detect with conventional methods. The electrical characteristics of the transistors varied depending on the maturation degree of the cell, which allowed for determining the best moment of astaxanthin production [4] (Figure 16).

#### 4.2.2. Organic Field-Effect Transistors

The functioning principles that govern the organic field effect transistors are the same as for the inorganic ones, and have been already recalled above (Section 4.1.1). One of the advantages of OFETs as bioelectronic substrates is that their electrically active surface is an organic film onto which the detection of electrical bio-signals is intrinsically easy. For using OFETs in cell cultures, and more particularly for cultivating cells on top of such devices, it is necessary to use organic semiconductors that are not toxic for the cells themselves, to allow attachment and growing. The chemical modification of organic surfaces, to make them biocompatible, is expected to be easier than for inorganic materials.

Even without the direct application in FET devices, the growth of living cells on organic semiconductors have been demonstrated (e.g., on pentacene) [85]. In practice, mouse neural cells’ growth on pentacene showed that the latter is stable upon prolonged contact with a physiological buffer and that the cells adhere and remain viable on it for more than 15 days. The authors succeeded in improving the adhesion by immobilizing laminin and poly-l-lysine on pentacene. Another article [86] reported, later, the success of the growth of stem cells on organic semiconductors, such as T6 (sexithiophene) and PDI-8CN2 (*N*,*N*’-bis(n-octyl)-dicyanoperylenediimide). As far as we know, further electrical characterizations of such cells@pentacene-based OFETs, cells@PDI8CN2, or cells@T6 have not been reported so far.

**Figure 17:**
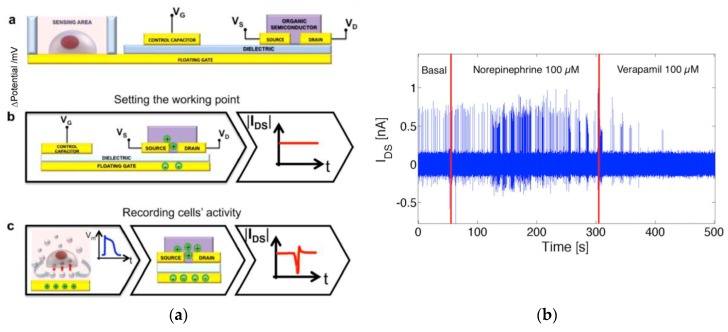
(**a**) Principle of the organic charge-modulated FET (OCMFET) (cross-section and working principles). The charge fluctuation over the sensing area determines the charge re-distribution inside the floating gate, which modulates the charge carriers’ density inside the channel of the transistor; (**b**) rat cardiomyocytes activity maintained at 37 °C for 8 days and measured with an OCMFET. The device is able to evidence that cell activity is accelerated by the addition of norepinephrine or attenuated with verapamil. Adapted from Spanu et al. [12], under the Creative Commons Attribution License (CC BY 4.0).

The contact of aqueous and saline media on organic semiconductors usually leads, after a short time, to the degradation of the electrical characteristics. Also, organic thin film transistors usually need to be operated at voltages over ten volt (which limits their use in aqueous solutions), and charge carrier mobility in organic semiconductors is generally several orders of magnitude smaller than in the inorganic ones, which limits the frequency range that can be explored. For this reason, Spanu et al. developed an organic charge-modulated FET (OCMFET), for which a floating gate, in contact with the living cells, avoids any contact between the culture medium and the organic semiconductor (Figure 17a) [12]. With such a device, the authors detected action potentials in physiological conditions from cardiac cells. The authors also showed that transduction acts through capacitive coupling between the floating gate and the cellular membrane. As shown in Figure 17b, action potentials were recorded with a high time resolution. This work also gave rise to a world patent [87].

**Figure 18:**
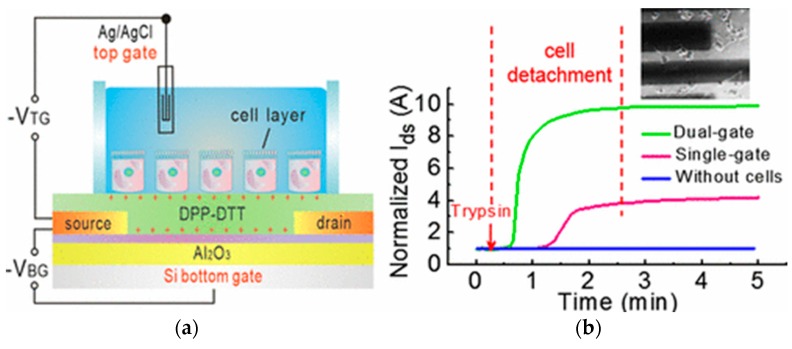
(**a**) Architecture of the dual-gate transistor, with a Ag/AgCl top gate and cells attached to the DPP-DTT semiconductor (poly[2,5-(2-octyldodecyl)-3,6-diketopyrrolopyrrole-alt-5,5-(2,5-di(thien-2-yl)thieno[3,2-b]thiophene)]; (**b**) time-dependent drain current curves showing cells detachment upon addition of trypsin. Reprinted with permission from Zhang et al. [88]. Copyright © 2017 American Chemical Society.

Liquid electrolyte-gated organic field effect transistors for cell monitoring are reviewed in the next section, but the work of Zhang et al. [88] can be considered in this section, because the architecture of their device was based on a dual gate (i.e., in-between the classical bottom-gated OFET and the EGOFET). In this work, the bottom gate was used in order to shift the operating domain within the range where the transconductance reaches its maximum (Figure 18). Indeed, such devices can be limited by their operation voltage; in other words, the voltage range where their transconductance is maximum could exceed the gate voltage window for which the gate current stays negligible. This gate voltage can be kept within a tolerable range if a voltage is, at the same time, imposed from another bottom, isolated, gate. For example, in this work, the authors were able to set the maximum transconductance point to a top-gate voltage below 0.3 V.

#### 4.2.3. Electrolyte-Gated Organic Field Effect Transistors

EGOFETs [89,90] have not been often used for cell monitoring, probably because they are more recent devices and have been much less described than OECTs. However, electrolyte-gated OFETs have attracted considerable attention for biosensing applications, for two main reasons: (1) the dielectric layer separating the gate electrode and the organic semiconductor in traditional OFETs is replaced by an electrolytic solution, which makes these transistors particularly suitable for liquid sample analysis; (2) the electrolyte leads to extremely high capacitances between the gate electrode and the semiconductor (up to a few hundreds of μF cm^−2^), which is reflected in the very small polarization voltages necessary to bias these devices (ca. 200–400 mV). Such low voltages are ideal for measurements in biological aqueous media and strongly reduce power consumption. By properly functionalizing the gate electrode with specific molecules (e.g., antibodies), the target molecules present inside the electrolyte interact with the gate surface, leading to modification of the interface capacitance which, in turn, modulates the drain current, even for an uncharged target at trace levels [91]. Their pertinence has been demonstrated for pH sensing [92,93] and biosensing for the detection of small toxic organic molecules [91,94] or proteins secreted by cells (e.g., interleukins [95,96] and TNF-α (tumor necrosis factor alpha) [97]). The fact that EGOFETs have not yet been used for living cells’ monitoring could be due to the fact that these EGOFETs have always been reported using top-gate configurations, and never using side-gates. So far, no examples of fully printed EGOFETs have been reported [98], which may be another factor that prevented their utilization in the field of cell monitoring.

However, Cramer and colleagues reported a work in 2013, where liquid gated organic FETs were used to monitor cells’ activity [99]. They used a pentacene-based EGOFET for monitoring the neuronal network activity. These devices are actually highly sensitive to small potential changes in the cell medium. Their devices did not record neurons independently, but several hundreds to thousands of neurons immobilized together on a single gate. They mostly demonstrated that pentacene enables good cellular adhesion, and that adhesion can be monitored through the coupling of the ionic currents at the cells–pentacene interface, with changes in OFET current.

## 5. Conclusions

There are three essential parts in a field-effect transistor: the gate, acting as the current switch; the dielectric, which is an electrolyte in several configurations; and the semiconductor. A number of the aforementioned devices are already hybrid systems, where one can consider that the cells play the role of a “living” electrolyte or “living” gate. For example, Tarabella et al., 2015 [100], described an OECT were the electrolyte above the PEDOT:PSS was replaced by a *Physarum polycephalum* cell, into the membrane of which the gate electrode has been plunged. It was shown that the cell acts as a reservoir of cations, which can be exchanged with the underlying PEDOT:PSS film, as in the case of an electrolyte; it is assumed that the cations in the intracellular matrix can cross the cell membrane through the ion channels, which open under the proper polarization of the gate. A large number of reported work also focusses on the quality of the contact between the cells and the underlying semiconductor, to create the most intimate contact between the cell membrane and the SC. However, as far as we know, FET devices where the cells themselves (collection of cells or single cell) totally replace the semiconductor have never been described to date, but would constitute a major achievement. In-between, the in-situ production of semiconductors by living cells, for example by oxidative polymerization of conjugated monomers through the action of reactive oxygen species (ROS) produced by the cells’ metabolism, has just started to be investigated and is very promising [101].
